# Safety of and Adverse Reactions to the COVID-19 Vaccine Among Pregnant and Breastfeeding Women

**DOI:** 10.3390/medsci13020038

**Published:** 2025-04-01

**Authors:** Nguyen Thi Minh Thanh, Le Thi Hang, Mai Trong Hung, Tran Hoa Phuong, Nguyen Thi Phuong Lan, Mac Dang Tuan, Nguyen Xuan Bach, Nguyen Duy Anh

**Affiliations:** 1 Expert Examination Department, Hanoi Obstetrics & Gynecology Hospital, Hanoi 100000, Vietnam; caophena2010@gmail.com; 2 Faculty of Medicine, VNU University of Medicine and Pharmacy, Hanoi 100000, Vietnam; hang.ump@vnu.edu.vn; 3 Hanoi Obstetrics & Gynecology Hospital, Hanoi 100000, Vietnam; bacsymaitronghung@gmail.com; 4 Training and Direction of Healthcare Activities Center, Hanoi Obstetrics & Gynecology Hospital, Hanoi 100000, Vietnam; tranhoaphuong80@gmail.com; 5 Faculty of Basic Science in Medicine and Pharmacy, VNU University of Medicine and Pharmacy, Hanoi 100000, Vietnam; phuonglan.ump@vnu.edu.vn; 6 Faculty of Public Health, VNU University of Medicine and Pharmacy, Hanoi 100000, Vietnam; tuanmac.smp@gmail.com; 7 Faculty of Pharmacy, VNU University of Medicine and Pharmacy, Hanoi 100000, Vietnam; 8 National Hospital of Obstetrics and Gynecology, Hanoi 100000, Vietnam; nguyenduyanh.nhog@gmail.com

**Keywords:** COVID-19, vaccines, pregnancy, breastfeeding, adverse reactions

## Abstract

Objectives: This study aimed to evaluate the incidence of adverse reactions to the COVID-19 vaccine among pregnant and breastfeeding women and identify associated demographic and clinical factors. Methods: A cross-sectional study was conducted at a hospital in Hanoi, Vietnam, from November 2021 to March 2022. A total of 1204 participants, including 991 pregnant women beyond 13 weeks of gestation and 213 breastfeeding women, were recruited through convenience sampling. Data were collected using a self-administered questionnaire designed to capture demographic information and adverse reactions occurring within seven to 28 days post-vaccination. Statistical analyses, including chi-square tests, Fisher’s exact tests, and logistic regression, were performed using Stata 16.0, with the significance set at *p* < 0.05. Results: The most common adverse reactions were localized pain at the injection site (26.2%), dizziness and fatigue (19.2%), and fever below 39 °C (29.1%). Severe adverse reactions, such as a tight throat, coma, and premature birth, were rare. A multivariate analysis identified the significant factors associated with the adverse reactions, including age (aOR = 2.04 for participants aged 36–40 years), occupation (lower odds for farmers and business professionals), urban residency (aOR = 0.64), and a history of allergies (aOR = 1.59). Education level, number of children, and gestational age were not significantly associated with adverse events. Conclusions: The findings support the safety of the COVID-19 vaccine in pregnant and breastfeeding women, with most of the adverse reactions being mild and self-limiting.

## 1. Introduction

Coronaviruses (CoVs) constitute a diverse family of RNA viruses capable of infecting both animals and humans. Among these, SARS-CoV-2—the causative agent of COVID-19—is primarily transmitted through respiratory droplets and, to a lesser extent, via contact with contaminated surfaces. The clinical spectrum of COVID-19 ranges from asymptomatic or mild symptoms to severe complications, including pneumonia, acute respiratory distress syndrome (ARDS), sepsis, multi-organ failure, and even death. Given these risks, pregnant and breastfeeding women are considered particularly vulnerable. Indeed, evidence suggests an elevated rate of severe complications and mortality in these groups; one study reported that two of 22 pregnant patients with severe pneumonia died from COVID-19 [1]. A systematic review and meta-analysis further confirmed that SARS-CoV-2 exposure during pregnancy is associated with adverse outcomes for both the mother and the infant [2].

Currently, there is no definitive cure for COVID-19. Therapeutic strategies worldwide have focused primarily on symptom management and the prevention of disease progression [3,4]. As a result, prophylactic measures—especially vaccination—are essential to public health, particularly for high-risk groups, such as pregnant and breastfeeding women [5]. The COVID-19 vaccine has demonstrated strong effectiveness in reducing morbidity and mortality related to the virus [6,7,8]. A systematic review and meta-analysis evaluating the vaccine’s effects in women before or during pregnancy showed that vaccination effectively reduces SARS-CoV-2-related complications [4]. Moreover, mounting evidence supports the safety of COVID-19 vaccination during pregnancy, with the benefits extending to fetuses and newborns through the passive transfer of maternal antibodies. Studies have consistently shown that vaccination does not increase the risk of adverse pregnancy or perinatal outcomes compared to unvaccinated pregnant women. For instance, a systematic review and meta-analysis of 67 studies involving 1,813,947 women found no association between COVID-19 vaccination during pregnancy and the increased risks of hypertensive disorders, caesarean section, or neonatal intensive care unit admissions [4]. Similarly, another systematic review concluded that COVID-19 vaccination during pregnancy is not associated with significant health risks for the mother, fetus, or newborn [9].

Despite these encouraging findings, pregnant and breastfeeding women were largely excluded from early COVID-19 vaccine clinical trials, particularly those involving messenger RNA (mRNA) vaccines. This lack of targeted data has contributed to vaccine hesitancy among these populations [10,11]. In light of this gap, the present study aims to evaluate the incidence of adverse reactions to the Pfizer/BioNTech COVID-19 vaccine among pregnant and breastfeeding women and to identify the demographic and clinical factors associated with these reactions.

## 2. Materials and Methods

### 2.1. Study Design and Setting

This cross-sectional study was conducted at a hospital in Hanoi, Vietnam, between November 2021 and March 2022. The study population included pregnant women beyond 13 weeks of gestation and breastfeeding women who had received the Pfizer/BioNTech COVID-19 vaccine at the hospital. Individuals presenting with acute illnesses were excluded from participation. A convenience sampling method was used to enroll a total of 1204 participants, comprising 991 pregnant women and 213 breastfeeding mothers.

### 2.2. Data Collection Method

A self-administered questionnaire was developed based on the guidelines for self-monitoring health following COVID-19 vaccination issued by the Ministry of Health of Vietnam and the Centers for Disease Control and Prevention (CDC). The questionnaire consisted of two sections: (1) general information, including demographic data and medical history, and (2) a list of potential adverse reactions. The reported symptoms included numbness around the lips or tongue; skin reactions such as rash, redness, purple rash, and bleeding; and cardiovascular, respiratory, digestive, and neurological symptoms, including severe pain and high fever. The participants were asked to self-report any adverse reactions that occurred within seven to 28 days after vaccination.

Before deployment, a questionnaire was piloted with ten participants to ensure clarity and user-friendliness. Feedback was used to refine and improve the questions. The finalized questionnaire was distributed through the hospital’s automated messaging system, which included a Google Form link. The participants were required to review the study introduction and indicate informed consent by selecting a designated checkbox before completing the form.

### 2.3. Statistical Analysis

All statistical analyses were conducted using Stata version 16.0. Descriptive statistics (frequencies, percentages, means, and standard deviations) were used to summarize the participants’ demographic and clinical characteristics. To compare the categorical variables between groups, we used the chi-square test when expected cell counts were sufficient and Fisher’s exact test when sample sizes were small or the expected frequencies were below 5, ensuring appropriate statistical validity. To explore the associations between the participant characteristics and the occurrence of adverse reactions, we first performed a univariate logistic regression analysis to estimate the crude odds ratios (cORs). The variables with a *p*-value < 0.20 in the univariate analysis were subsequently included in a multivariate logistic regression analysis to control for potential confounding factors and estimate the adjusted odds ratios (aORs). A two-tailed *p*-value of < 0.05 was considered statistically significant in the final model. This approach was chosen to identify the independent predictors of the adverse reactions while accounting for the influence of covariates.

## 3. Results

Table 1 presents the demographic and clinical characteristics of the study participants. The majority were aged 26–30 years (44.8%), with a mean age of 30.25 ± 4.36 years (range: 19–44 years). Most of the participants had attained university or postgraduate education (68.3%), while 21.8% had completed high school. In terms of occupation, the majority were farmers (66.0%), followed by individuals engaged in business activities (21.6%). A significant proportion of the participants resided in rural areas (88.1%) compared to urban areas (11.9%). The prevalence of allergies was relatively low, with 90.5% of the participants reporting no history of allergic conditions. Regarding family structure, 45.8% had one child, 26.1% had two children, and 21.1% were childless. Most of the participants (92.9%) had received two doses of the COVID-19 vaccine, while only 7.1% had received a single dose.

Table 2 summarizes the symptoms reported by pregnant and breastfeeding women following COVID-19 vaccination. The most frequently reported symptoms were pain, soreness, and swelling at the injection site, experienced by 26.0% of pregnant women and 26.2% of breastfeeding women. Vestibular symptoms, such as dizziness and fatigue, were also common, reported by 10.6% of pregnant and 12.1% of breastfeeding participants, accounting for 19.2% of all cases. Fever below 39 °C was reported by 12.9% of pregnant women, 15.8% of breastfeeding women, and 29.1% overall. Other common symptoms included prolonged headaches (6.1% in pregnant and 6.2% in breastfeeding women) and chest pain (4.4% and 4.2%, respectively). Severe adverse reactions, such as coma, a tight throat, and premature birth, were rare across both groups.

Table 3 presents the results of the univariate and multivariate logistic regression analyses. Participants aged 36–40 years were significantly more likely to report adverse reactions compared to the reference group aged 18–25 years (adjusted odds ratio [aOR] = 2.04, 95% confidence interval [CI]: 1.10–3.78, *p* < 0.05), suggesting that advancing maternal age may be associated with heightened immune reactivity or increased sensitivity. Occupation also showed a significant association. Farmers (aOR = 0.56, 95% CI: 0.34–0.95, *p* < 0.05) and business workers (aOR = 0.52, 95% CI: 0.30–0.91, *p* < 0.05) had significantly lower odds of adverse reactions compared to freelancers or individuals performing housework. Similarly, urban residency was associated with a lower likelihood of experiencing adverse reactions compared to rural residency (aOR = 0.64, 95% CI: 0.43–0.97, *p* < 0.05). A history of allergies was another strong predictor, with the participants who reported allergies being more likely to experience adverse reactions (aOR = 1.59, 95% CI: 1.02–2.51, *p* < 0.05). Other variables, including education level, number of children, and gestational age, were not significantly associated with adverse reactions in the adjusted analysis.

## 4. Discussion

This study provides valuable insights into the demographic and clinical characteristics of pregnant and breastfeeding women following COVID-19 vaccination, as well as the nature and frequency of the adverse reactions and the associated influencing factors. The findings highlight both the prevalence of adverse events and the demographic and clinical variables contributing to their occurrence.

The adverse reactions reported in this study were predominantly mild and consistent with the existing literature, including localized pain at the injection site, fatigue, headache, dizziness, and low-grade fever. These symptoms were generally self-limiting and manageable within routine clinical care. Severe adverse events, such as a tight throat, coma, and premature birth, were rare, reinforcing the safety of COVID-19 vaccination in pregnant and breastfeeding populations. The incidence of any adverse reaction (52.0%) was slightly lower than that reported in previous studies by Perh (55.9%) and Czudy (54.8%), and notably lower than Beatty’s findings (80.3%) [12,13,14]. Variations in immune responses across ethnic groups may account for these differences [15]. These findings are supported by Silvia et al. (2024), who reported injection site pain in up to 77% of participants [4], and by other studies confirming the transient nature of systemic symptoms such as fatigue, headache, and myalgia [16,17].

Gastrointestinal symptoms such as vomiting and diarrhea were reported by 4.5% of participants, a rate comparable to that found in Bertrand et al.’s study, which recorded diarrhea in 3.4% and vomiting in 2.9% of cases, and by Shimabukuro et al. who reported rates of 5% and 4.5%, respectively [18,19]. Adverse events with potential fetal implications—including epistaxis, abdominal pain and bleeding, premature birth, delayed fetal development, and loss of breast milk—were extremely rare, occurring at a rate of just 0.1%. These findings are in line with those of Shimabukuro et al. who reported no cases of miscarriage, stillbirth, or congenital abnormalities, and with Bertrand et al. who documented an 11.4% reduction in breast milk supply among breastfeeding women [18,19]. Further supporting vaccine safety, a large study by Kachikis et al. (2022) involving 17,014 participants found that 97.6% of pregnant women reported no obstetric or lactation concerns post-vaccination [20]. Similarly, Covas et al. (2023) reported an adverse event incidence rate of 309.4 per 100,000 doses in Brazil, with the vast majority being non-severe [21]. Among women with autoimmune diseases, a COVAD study indicated that 17.5% of pregnant participants experienced post-vaccination disease flares, which were not more frequent than those in non-pregnant counterparts and were generally well-managed with glucocorticoids or immunosuppressive adjustments [22].

This study identified several factors significantly associated with the adverse reactions following COVID-19 vaccination among pregnant and breastfeeding women. Age emerged as a key factor, with participants aged 31–35 and 36–40 years being 1.64 to 2.23 times more likely to experience adverse reactions compared to those aged 18–25 years. This association was confirmed through both univariate and multivariate logistic regression analyses, suggesting that age may influence vaccine tolerance. Similar findings were reported by Fernando et al., who observed an increased risk of adverse reactions among individuals under 55 years of age, likely due to heightened immune responses to vaccination [23,24]. Although education level was not significantly associated with adverse reactions, occupation was found to be an influential factor. Workers and individuals in business roles were less likely to report adverse events compared to freelancers or houseworkers. Multivariate analysis further indicated that farmers and business professionals had a reduced risk of adverse reactions. These results are in line with the study by Nguyen Hoang An et al., which found that individuals performing lighter work experienced fewer adverse reactions after receiving the AstraZeneca vaccine [12].

Urban residency was associated with a lower risk of adverse reactions compared to rural residency, possibly due to better access to healthcare services and safer vaccination conditions in urban settings. Additionally, a history of allergies significantly increased the risk of adverse reactions, consistent with the findings from Lily et al. and Gang Chen et al., who identified a history of allergies as a strong predictor of vaccine-related hypersensitivity reactions, including a rash and anaphylaxis [25,26]. While women with two or three children were more likely to report adverse reactions in the univariate analysis, this association was not confirmed in the multivariate model, suggesting that further research is needed to explore the underlying mechanisms. Gestational age was not significantly associated with adverse reactions, aligning with previous studies that reported no relationship between gestational age and vaccine-related adverse events [27]. These findings underscore the importance of considering individual demographic and clinical characteristics when counseling and monitoring pregnant and breastfeeding women during vaccination, while reinforcing the overall safety of COVID-19 vaccines in this population.

The implications of this study are multifaceted and highly relevant to public health efforts. First, the findings reinforce the favorable safety profile of COVID-19 vaccines among pregnant and breastfeeding women, which is critical for promoting vaccine acceptance in these high-risk groups. Second, the identification of the specific factors associated with adverse reactions, such as age, occupation, allergy history, and place of residence, can guide the development of targeted counseling and monitoring strategies, improving vaccine delivery and safety outcomes. Third, these results provide evidence-based reassurance that can be leveraged in public health messaging to address concerns and reduce vaccine hesitancy. By emphasizing the predominantly mild and self-limiting nature of the adverse reactions, healthcare providers and policymakers can build trust and encourage informed decision-making among pregnant and breastfeeding individuals, thereby supporting broader vaccination efforts and maternal–infant health protection.

This study has several limitations that should be acknowledged. First, the cross-sectional design limits the ability to establish causal relationships between the demographic characteristics and adverse reactions. Additionally, the reliance on self-reported symptoms may introduce recall bias and affect the accuracy of adverse event classification. The use of convenience sampling may limit the representativeness of the sample and reduce the generalizability of the findings to broader populations. Moreover, several potentially important confounding variables, such as pre-existing medical conditions, body mass index (BMI), diet, smoking status, physical activity, and socioeconomic factors, were not collected, which may have influenced immune responses and reported outcomes. Furthermore, data verification through clinical records was not feasible as this study relied on participant-reported responses. Although it would have been valuable to analyze the adverse reactions separately by vaccine dose, the distribution of participants in our study was highly imbalanced, with 92.9% having received two doses and only 7.1% having received a single dose. This limited the statistical power and reliability of a dose-specific comparison. To avoid potentially misleading conclusions, we did not conduct a separate analysis by dose. Finally, future research should incorporate more rigorous sampling methods, objective data verification, long-term follow-up, and comprehensive confounder control to enhance the robustness and generalizability of the findings, and provide a more complete understanding of COVID-19 vaccine safety in pregnant and breastfeeding populations.

## 5. Conclusions

The findings of this study support the safety of COVID-19 vaccines in pregnant and breastfeeding women, with most of the adverse reactions being mild and self-limiting. Age, occupation, urban residency, and a history of allergies were identified as significant factors associated with the occurrence of adverse events. These results highlight the importance of personalized counseling and careful monitoring during vaccination to enhance vaccine confidence and ensure the safety of these vulnerable populations. Further research is needed to address this study’s limitations and to explore the long-term outcomes in this population.

## Figures and Tables

**Table 1:** Demographic and clinical characteristics of the participants.

Characteristics		N	%
Age	18–25	139	11.5
	26–30	539	44.8
	31–35	379	31.5
	36–40	124	10.3
	>40	23	1.9
	Mean ± SD (Min–Max)	30.25 ± 4.36 (19–44)	
Education	Elementary school or less	30	2.5
	Junior high school	90	7.5
	High school	262	21.8
	University/post-graduate	822	68.3
Job	Freelancers/housework	89	7.4
	Farmer	795	66.0
	Worker	36	3.0
	Business	260	21.6
	Officer	13	1.1
	Unemployment	11	0.9
Residence	Rural	1061	88.1
	Urban	143	11.9
History of allergies	No	1090	90.5
	Yes	114	9.5
Number of children	0	254	21.1
	1	552	45.8
	2	314	26.1
	3	78	6.5
	4	6	0.5
Number of injections	1	85	7.1
	2	1119	92.9

**Table 2:** Symptoms reported by pregnant and breastfeeding women following COVID-19 vaccination.

Group	Symptoms	Pregnant Women (n = 991)	Breastfeeding Women (n = 213)	Total (n = 1204)
		n (%)	n (%)	n (%)
**Mouth**	Numbness around the lips/tongue	9 (0.9%)	17 (1.4%)	8 (3.8%)
**Skin**	Rash	2 (0.2%)	3 (0.2%)	1 (0.5%)
	Redness, purple rash	7 (0.7%)	8 (0.7%)	1 (0.5%)
	Bleeding	16 (1.6%)	23 (1.9%)	7 (3.3%)
**Throat**	Itchy throat	1 (0.1%)	4 (0.3%)	3 (1.4%)
	Tight throat	1 (0.1%)	1 (0.1%)	0 (0.0%)
	Choking, difficulty speaking	21 (2.1%)	26 (2.2%)	5 (2.3%)
**Nervous system**	Prolonged, severe, lethargic headache	60 (6.1%)	75 (6.2%)	15 (7.0%)
	Drowsiness, confusion	1 (0.1%)	3 (0.2%)	2 (0.9%)
	Coma	1 (0.1%)	1 (0.1%)	0 (0.0%)
**Heart**	Chest pain	44 (4.4%)	51 (4.2%)	7 (3.3%)
	Nervous palpitations	3 (0.3%)	6 (0.5%)	3 (1.4%)
	Fainting	1 (0.1%)	1 (0.1%)	0 (0.0%)
**Digestion**	Vomiting, diarrhea	45 (4.5%)	54 (4.5%)	9 (4.2%)
**Respiratory**	Shortness of breath	35 (3.5%)	38 (3.2%)	3 (1.4%)
	Wheezing	4 (0.4%)	4 (0.3%)	0 (0.0%)
**Vestibular disorders**	Dizziness, lightheadedness, unusual fatigue	105 (10.6%)	146 (12.1%)	41 (19.2%)
**Painful**	Pain, soreness, swelling, or itching at the injection site or the entire body	258 (26.0%)	316 (26.2%)	58 (27.2%)
**Fever**	High fever (≥39 °C)	0 (0.0%)	3 (0.2%)	3 (1.4%)
	Fever (<39 °C)	128 (12.9%)	190 (15.8%)	62 (29.1%)
**Other**	Epistaxis	1 (0.1%)	1 (0.1%)	0 (0.0%)
	Abdominal pain and bleeding	1 (0.1%)	1 (0.1%)	0 (0.0%)
	Premature birth	0 (0.0%)	1 (0.1%)	1 (0.5%)
	Slow fetal development	1 (0.1%)	1 (0.1%)	0 (0.0%)
	Loss of breast milk	0 (0.0%)	1 (0.1%)	1 (0.1%)
	Axillary/neck lymph nodes	3 (0.3%)	3 (0.3%)	0 (0.0%)
	Flu-like symptoms (runny nose, sneezing)	8 (0.8%)	12 (1.0%)	4 (2.0%)

**Table 3:** Univariate and multivariate logistic regressions to identify the factors associated with adverse events.

Factor		Adverse Reaction, n (%)		cOR	*p* (95%CI)	aOR	CI 95%
		No	Yes				
Age	18–25	79 (56.8)	60 (43.2)	reference		reference	
	26–30	271 (50.3)	268 (49.7)	1.30	0.168 (0.89–1.90)	1.32	0.85–2.06
	31–35	169 (44.6)	210 (55.4)	1.64	0.014 (1.11–2.42)	1.56	0.96–2.52
	36–40	46 (37.1)	78 (62.9)	2.23	0.001 (1.36–3.66)	2.04 *	1.10–3.78
	>40	13 (56.5)	10 (62.9)	1.01	0.978 (0.41–2.47)	0.81	0.27–2.42
Education	Elementary school or less	16 (53.3)	14 (46.7)	reference		reference	
	Junior high school	50 (55.6)	40 (44.4)	0.91	0.832 (0.40–2.10)	1.03	0.39–2.69
	High school	141 (53.8)	121 (46.2)	0.98	0.960 (0.46–2.09)	1.25	0.51–3.12
	University/post-graduate	371 (45.1)	451 (54.9)	1.39	0.378 (0.67–2.88)	1.48	0.59–3.65
Job	Freelancers/housework	32 (36.0)	57 (64.0)	reference		reference	
	Farmer	372 (46.8)	423 (53.2)	0.64	0.053 (0.41–1.01)	0.56 *	0.34–0.95
	Worker	21 (58.3)	15 (41.7)	0.40	0.024 (0.18–0.89)	0.45	0.18–1.10
	Business	143 (55.0)	117 (45.0)	0.46	0.002 (0.28–0.76)	0.52 *	0.30–0.91
	Officer	5 (38.5)	8 (61.5)	0.90	0.861 (0.27–2.98)	1.12	0.30–4.14
	Unemployment	5 (45.5)	6 (54.5)	0.67	0.540 (0.19–2.38)	0.69	0.14–3.54
Residence	Rural	493 (46.5)	568 (53.5)	reference		reference	
	Urban	85 (59.4)	58 (40.6)	0.59	0.004 (0.42–0.85)	0.64 *	0.43–0.97
History of allergies	No	534 (49.0)	556 (51.0)	reference		reference	
	Yes	44 (38.6)	70 (61.4)	1.53	0.036 (1.03–2.27)	1.59 *	1.02–2.51
Number of children	0	132 (52.0)	122 (48.0)	reference		reference	
	1	288 (52.2)	264 (47.8)	0.99	0.957 (0.74–1.34)	0.88	0.64–1.22
	2	127 (40.4)	187 (59.6)	1.59	0.006 (1.14–2.22)	1.21	0.80–1.82
	3	29 (37.2)	49 (62.8)	1.83	0.023 (1.09–3.08)	1.57	0.74–3.32
	4	2 (33.3)	4 (66.7)	2.16	0.378 (0.39–12.03)	1.73	0.14–21.33
Gestational age	2nd-trimester pregnancy	157 (48.3)	168 (51.7)	reference		reference	
	3rd-trimester pregnancy	342 (51.4)	324 (48.6)	0.89	0.368 (0.68–1.15)	0.91	0.69–1.19

* *p* < 0.05; cOR = crude odd ratios; aOR = adjusted odd ratios.

## Data Availability

Data are available upon request.
